# Natural Resistance to HIV Infection: Role of Immune Activation

**DOI:** 10.1002/iid3.70138

**Published:** 2025-02-25

**Authors:** María M. Naranjo‐Covo, Daniel S. Rincón‐Tabares, Lizdany Flórez‐Álvarez, Juan C. Hernandez, Wildeman Zapata‐Builes

**Affiliations:** ^1^ Grupo Inmunovirología, Facultad de Medicina Universidad de Antioquia Medellín Colombia; ^2^ Grupo Infettare, Facultad de Medicina Universidad Cooperativa de Colombia Medellín Colombia; ^3^ Departamento de Parasitología, Instituto de Ciencias Biomédicas Universidad de Sao Paulo Sao Paulo Brazil

**Keywords:** HESN, HIV‐1, immune activation, natural resistance

## Abstract

**Introduction:**

Although repeated exposure to HIV‐1 can result in infection, some individuals remain seronegative without clinical or serologic evidence of infection; these individuals are known as HIV‐1‐exposed seronegative individuals. This population has been extensively studied to understand the mechanisms associated with natural resistance to HIV infection. Two main hypotheses have been proposed to explain this resistance: some researchers associated resistance with a low activation phenotype characterized by a decrease in the activation and proliferation of immune system cells linked with infection control and decreased production of cytokines and pro‐inflammatory molecules, whereas others suggest that resistance is related to immune system activation and the expression of high levels of chemokines, pro‐inflammatory cytokines and antiviral molecules.

**Aims:**

Our study aims to review and analyze the most relevant evidence supporting the role of the activation level of the immune system during natural resistance to HIV‐1 infection.

**Methods:**

A search was conducted via the PubMed, SciELO and ScienceDirect databases. The literature search was performed in a nonsystematic manner. Articles published in the last five decades addressing immune activation mechanisms in natural resistance to HIV were reviewed.

**Results:**

A low‐activation phenotype, characterized by a high frequency of Treg cells; reduced expression of CD25, CD38, and HLA‐DR; and lower production of pro‐inflammatory cytokines in peripheral and mucosal tissues, plays a key role in reducing the number of activated cells susceptible to infection, but it minimizes chronic inflammation, facilitating viral entry and spread. In contrast, the activation phenotype is associated with high expression of markers such as CD25, CD38, and HLA‐DR, along with elevated high levels of interferon‐stimulated genes and pro‐inflammatory cytokines. This profile could promote infection control while increasing the number of virus‐susceptible cells.

**Conclusion:**

The complexity of the immune response during HIV exposure, reflected in the conflicting evidence concerning whether low or high immune activation offers protection against infection, suggests that there may be multiple pathways to HIV‐1 resistance, influenced by factors such as the type of viral exposure, the immune environment, and individual genetics. Further research is needed to determine which immune states are protective and how these responses can be modulated to prevent infection.

## Graphical abstract

High and low immune activation have been proposed as a possible explanation in HIV‐1 resistance. This review focuses on the most relevant evidence supporting each perspective.

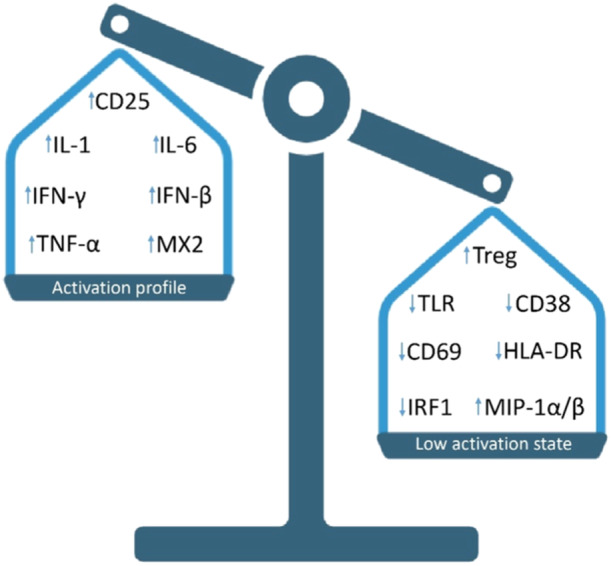

## 1 Introduction

Repeated contact with human immunodeficiency virus (HIV‐1) can lead to infection; however, some individuals remain uninfected despite numerous high‐risk exposures or repeated high‐risk behaviors in areas with a high HIV‐1 prevalence. This population is known as HIV‐1‐exposed seronegative (HESN) individuals, and they are important for studying potential factors mediating natural resistance to this infection [1, 2]. HESNs can be categorized into three major groups: (i) serodiscordant couples (a couple where one partner is HIV‐positive and the other is HIV‐negative); (ii) individuals engaged in high‐risk sexual behaviors, such as commercial sex workers (CSWs) and men who have sex with men (MSM); and (iii) individuals exposed to HIV through nonsexual means, including injection drug users (IDUs), infants born to HIV‐positive mothers, hemophiliacs, and others who have come into contact with contaminated blood products [3]. CSWs and MSM with high‐risk sexual behavior constitute an interesting group for the study of natural resistance to HIV‐1 infection because of the higher probability of cross‐reactive responses against different HIV‐1 quasispecies than groups with low‐risk sexual behavior, such as serodiscordant couples in stable relationships [3]. Therefore, studying these groups could lead to the development of therapeutic alternatives or vaccines that control HIV‐1 transmission.

Since 1984, when HIV‐1 was identified as the virus that causes acquired immunodeficiency syndrome (AIDS), multiple studies have evaluated natural resistance to infection [4, 5, 6]. Homozygosity for the CCR5Δ32 mutation is the most critical genetic mechanism of natural resistance to HIV‐1 infection. Homozygous individuals express a truncated protein, rendering cells resistant to HIV because the virus cannot fuse with the host cell surface, blocking entry [7, 8]. The frequency of the CCR5 Δ32 allele worldwide is approximately 3% [9]; however, this mutation explains only approximately 14.5% of the cases in the HESN population [10], suggesting the presence of underlying mechanisms associated with natural resistance to HIV‐1 infection.

In this regard, genome‐wide association studies (GWAS) have been instrumental in identifying genetic variants beyond CCR5Δ32 that may contribute to HIV‐1 resistance. Variants in genes such as HLA‐B and HLA‐C are linked to effective infection control by facilitating the presentation of viral peptides to T cells, thereby enhancing the immune response [11, 12, 13]. Other examples include functional variants of the APOBEC3G gene, an enzyme with antiviral activity capable of interfering with HIV replication [14, 15, 16], and genes related to HIV restriction, such as TRIM5α [17] and SAMHD1 [18]. However, the evidence for these is less consistent compared to HLA variants. Nonetheless, GWAS in HESN populations face challenges, including small cohort sizes and heterogeneity in inclusion criteria, which complicate the identification of robust associations. These limitations notwithstanding, such studies underscore the complexity of genetic mechanisms contributing to HIV‐1 resistance, highlighting a multifactorial phenomenon wherein multiple genes and biological pathways interact.

In addition, natural resistance to HIV‐1 points to the stimulation of various immune effector mechanisms that enable the immune system of HESN to handle HIV‐1, preventing the establishment of infection [19]. Specific HESN cohorts exhibit a specific T‐cell immune response against HIV [20], increased NK cell activity [21], and increased levels of soluble factors with anti‐HIV activity (i.e., beta‐defensins, beta‐chemokines, elafin/trappin‐2, APOBEC3G, TRIM5α, serpin, and cystatin antiproteases) in the circulation and genital mucosa, mainly at virus entry sites [22, 23, 24, 25, 26, 27]. Likewise, protection has been associated with other factors, such as HIV‐1‐specific IgA, which are plasma and genital tract antibodies that can neutralize primary HIV‐1 isolates in peripheral blood mononuclear cells (PBMCs) and prevent HIV‐1 transcytosis through human epithelial tissues in vitro [28, 29].

Interestingly, IL‐21, a cytokine with immunomodulatory properties, is capable of controlling HIV‐1 infection. A previous study conducted in a Colombian cohort of HESN patients revealed that their PBMCs were less susceptible to infection and expressed higher levels of IL‐21 mRNA than those of healthy controls at baseline and 7 days postinfection [30]. Additionally, IL‐21 has been demonstrated to influence HIV‐1 infection by activating various antiviral mechanisms, such as engaging the Th17 lineage and inducing microRNA (miR)‐29, which possesses anti‐HIV properties [31]. However, these resistance‐attributable mechanisms have not been explored in all HESN cohorts, and it is unknown whether they are present in each cohort.

In the last two decades, various studies have sought to explain the immunological features underlying the resistance observed in HESN. These studies have identified two main approaches that, despite their differences, provide valuable insights into how this resistance might be maintained.

The first approach suggests that HESN individuals exhibit an immunological quiescent or low‐activation phenotype. This state is characterized by low proliferation of CD4^+^ and CD8^+^ T cells; reduced levels of activation markers such as HLA‐DR, CD38, and CD70; and generally decreased secretion of effector molecules and pro‐inflammatory cytokines such as IL‐1β, IL‐6, colony‐stimulating factor 2 (CSF2), IFNγ, and TNF in the PBMCs (peripheral blood mononuclear cells) of these individuals. Interestingly, some of these mediators are produced at similar levels to those in healthy controls following antigenic stimulation, indicating that HESN cells remain functional [32, 33, 34]. In this context, the low‐activation phenotype could contribute to HIV‐1 resistance by maintaining immune cells in an unstimulated state, thus avoiding excessive inflammatory responses that might favor infection.

In contrast, the second approach describes an immune activation phenotype in HESN individuals with distinct immunological features, such as the expression of IL‐6, IL‐10, IFN‐γ, TNF‐α, Toll‐like receptors, APOBEC3G [35], α‐ and β‐defensins [22, 36], and chemokines such as CXCL12, CCL3, CCL4, and CCL5 in the peripheral blood, genitals, and oral mucosa. This phenotype appears to be more closely related to HIV‐infected subjects than to uninfected controls [37]. Thus, immune activation could be associated with specific resistance mechanisms to HIV‐1 and a more effective immune response against the virus. Despite the growing body of evidence supporting these hypotheses, controversy remains regarding the activation and low‐activation or quiescent phenotype in HESN individuals. Therefore, this review highlights the main differences in immune parameters between the two approaches and their possible implications for resistance and susceptibility to HIV‐1 infection and progression. The specific mechanisms described in HESN will subsequently be addressed to better understand how these mechanisms might contribute to HIV‐1 resistance.

## 2 Quiescence, Immune Quiescence, or a Low‐Activation Phenotype

A previous review used the term quiescence to refer to the low‐activation phenotype [38]; however, the term quiescence is defined as a cellular state characterized by prolonged and reversible entry into the G0 phase of the cell cycle [39]. This state is distinct from senescence or terminal differentiation, which involve irreversible exit from the cell cycle [40, 41]. This phenomenon has been observed in various cell populations, including tissue‐resident stem cells (hematopoietic, muscle, and neural) [42] and differentiated cells (fibroblasts [43] and lymphocytes) [44].

*In vitro* studies have demonstrated that HIV can infect “quiescent” CD4^+^ T cells, although viral replication is inefficient, leading to abortive infections [45, 46]. Several authors suggest that the host factors necessary for HIV‐1 replication are expressed primarily in activated cells [47, 48]. Despite advances in understanding the quiescent phenotype, controversies remain regarding the definition of this concept compared with the low‐activation phenotype and its implications for natural resistance to HIV‐1 infection [49, 50].

Historically, the quiescent phenotype was thought to represent an anergic state characterized by hyporesponsiveness to antigen recognition in the absence of costimulation, leading to impaired cell proliferation and cytokine production [51]. In contrast, a quiescent state involves transcriptional and metabolic control of activation signals, with cells able to respond to activation or exit quiescence upon stimulation [38, 52, 53, 54]. This state is distinct from the anergic state, which is induced by insufficient costimulation via CD28 or excessive coinhibitory signaling through CTLA‐4 and is characterized by low or absent IL‐2 production [55, 56]. In quiescent T cells, autocrine and paracrine IL‐2 production can be reestablished through CD28 costimulation [38].

Recent studies have linked natural resistance to HIV‐1 with quiescence, particularly in cells in the G0/1a phase, which are quiescent, whereas cells in the G1b phase are more permissive to HIV infection because of high RNA synthesis without DNA synthesis [45, 57, 58]. Advances in understanding quiescence have led to the use of markers such as the nuclear protein Ki‐67 to differentiate between proliferating and quiescent cells [59, 60]. Additionally, different phosphorylation patterns of the retinoblastoma protein (pRB) serve as transcriptional regulators of cell cycle progression, with pRB being phosphorylated in activated cells [59, 61]; further investigations through in vitro assays into the role of quiescence in natural resistance to HIV‐1 in various HESN cohorts would be valuable.

Quiescence in T cells is characterized by specific features: (i) entry into the G0 phase of the cell cycle; (ii) cessation of cell growth; (iii) inhibition of IL‐2 signaling; (iv) low nutrient uptake; (v) predominantly catabolic metabolism; and (vi) reprogramming of mitochondrial metabolism through the regulation of ATP production, cell survival, and biosynthesis [38]. In addition, Card et al. described immunological quiescence as a state defined by reduced T‐cell activation markers, downregulation of gene transcription, and reduced levels of cytokines and pro‐inflammatory molecules produced in the circulation and mucosa [62]; however, we believe these characteristics primarily reflect a low activation phenotype rather than true quiescence. Therefore, we use the term “low activation” to describe this immunological phenomenon as a protective correlate against HIV‐1 infection.

The low‐activation phenotype encompasses several mechanisms, including a regulatory immune response essential for understanding natural resistance to HIV‐1. Treg cells, for example, are crucial for maintaining the balance between overactive and immunosuppressive responses through the production of cytokines such as IL‐10, IL‐35 [63, 64], and transforming growth factor‐beta‐1 (TGF‐B1) [65], which contribute to sustained low activation. Studies have shown that Treg cells can control cytotoxic T lymphocyte responses in vivo by inhibiting T‐cell proliferation and clonal expansion [66].

In this sense, a study performed in participants from the Majengo commercial sex‐work cohort in Nairobi, Kenya, revealed that a greater frequency of Treg cells (CD4^+^CD25^+^FOXP3^+^) in peripheral blood could mediate the suppression of T‐cell activation, playing a crucial role in promoting an anti‐inflammatory state in these individuals [67]. However, instead of an anti‐inflammatory state, a tolerogenic process may occur, as happens during the immune response in the gut‐associated lymphoid tissue. To our knowledge, this phenomenon associated with tolerogenesis has not been studied in the HESN population.

Similarly, the expression of markers such as HLA‐DR and CD38 was strongly downregulated in CD4^+^ and CD8^+^ T cells from HESN individuals compared with those from HIV‐positive individuals [67]. Furthermore, although no statistically significant differences were observed, a lower percentage of CD4^+^ CD69^+^ T cells and CD8^+^ CD69^+^ T cells was observed in HESN women than in HIV‐positive individuals and healthy control individuals [67]. Another report in a Kenyan cohort of HESN CSW women (Pumwani cohort) revealed that these individuals produced lower levels of pro‐inflammatory cytokines than did HIV‐negative control individuals. It was also demonstrated that CD4^+^ T cells from the HESN cohort expressed lower levels of crucial genes for TCR signaling, where the reduced expression of these molecules reflects lower T‐cell activation [34]. This evidence suggests that a greater frequency of Treg cells, low levels of activation markers, and the differential expression of genes involved in low cellular activation could be natural resistance mechanisms to HIV‐1.

In the case of sexual exposure to HIV‐1, the epithelium works as the first barrier for HIV entry into the submucosa, showing variable structural conformations with different implications for viral transmission [68]. In this context, the anorectal epithelium has the highest likelihood of HIV transmission (0.3%–5%) compared with the female genital epithelium (0.05%–0.5%) and the male genital epithelium (0.04%–0.14%), with the oral mucosa being the least susceptible at 0.01% [69, 70, 71]. These dissimilarities could contribute to natural resistance in the HESN population through sexual contact. Similarly, in a study evaluating the cervicovaginal epithelium, lower levels of CD4^+^ CD69^+^ and CD8^+^ CD69^+^ T cells were found in HESN sex workers than in healthy controls; however, no significant differences were found in the ectocervical epithelial thickness, frequency of CD4^+^ CCR5^+^ cells, or levels of several pro‐inflammatory cytokines and chemokines in cervicovaginal lavages [50]. Although these studies evaluated CD69 expression as an activation marker, this molecule could also indicate a phenotype of tissue‐resident memory cells [72]. Therefore, to determine the context in which CD69 is active, it is important to consider the duration of expression, since CD69, as an activation marker, is observed a few hours after cellular activation; the context of stimulation, as the expression of this marker can be induced by activation signals such as specific antigens, cytokines, or mitogenic agents; and the simultaneous expression of other activation markers such as HLA‐DR, CD25, and CD38 [73, 74].

Some authors have proposed that a possible explanation for the difference in HIV‐1 acquisition is not only low activation at the cervical level but also the high production of soluble factors such as RANTES, MIP‐1α, and MIP‐1β, which are CC chemokines that bind to and activate the CCR5 chemokine receptor, thereby preventing the entry of HIV‐1 strains that rely on it as coreceptors [75]. In fact, higher levels of anti‐inflammatory antiproteases, such as serpins and cystatins, have been detected in HESN individuals than in HIV‐1‐positive and HIV‐1‐negative individuals [26, 76]. Similarly, another study evaluating the cervicovaginal lavage of HESN women revealed lower levels of pro‐inflammatory molecules such as IL‐1α, MIG and IP‐10 in the genital tract of HESN women than in the genital tract of HIV‐1‐positive and ‐negative individuals [77].

Interestingly, Fulcher et al. reported that in mucosal explants obtained from freshly collected colorectal biopsies of healthy controls and HESN individuals, which were stimulated with specific innate immune ligands and whole inactivated pathogens, there was a reduced production of pro‐inflammatory cytokines such as IL‐6 and IL‐1β through TLR4, TLR7, TLR9 and NOD2 but similar production of the cytokines IL‐10, IL‐4, and IL‐5 compared with that in healthy controls, suggesting that HESN individuals exhibit diminished immune reactivity, which reduces the number of activated cells susceptible to HIV‐1 infection [78].

Similarly, in the female genital tract of the HESN Pumwani cohort, which has an estimated at least 64 unprotected sexual exposures to HIV‐1 per year [2], the genes encoding TLR2, TLR4, TLR7, TLR8, RIG‐I, and MDA5 in cervical mononuclear cells presented reduced expression, but these cells were responsive to activation by the TLR7/8 ligand ssRNA40, which suggests that despite the low levels of these PRRs, HESN could activate an appropriate antiviral response after stimulation [79]. Additionally, highly significant increases in the mRNA expression of TLR1 and TLR10 were detected in primary bone marrow cells infected with HIV‐1 compared with those in uninfected bone marrow cells from Nigeria. These findings indicate that TLRs are strongly expressed in primary bone marrow cells and that this expression may be closely linked to an individual's HIV‐1 infection and innate immune response [80].

Interferon regulatory factor 1 (IRF1) is part of a broad family of transcription factors that are essential for various biological processes, such as antigen processing and presentation, NK cell activity, nitric oxide synthetase induction, and Th1 and Th2 differentiation, and functions as key mediators of innate and adaptive immune responses [81]. Previous studies have described three polymorphisms in *irf‐1* that appear to have a beneficial effect on natural resistance to HIV‐1 infection, where individuals with one or more of these polymorphisms presented lower IRF1 protein expression and reduced responsiveness to IFN‐ɣ stimulation; furthermore, these polymorphisms did not affect disease progression in terms of decreased CD4^+^ T cells and HIV‐1 viral load, suggesting that the protective effect is limited to the early stages of infection [82, 83]. In fact, Ji et al., using a model of HIV‐1 infection with PBMCs from individuals with different IRF1 genotypes, reported that IRF1 expression at 48 h and subsequent times in individuals with “protective genotypes” was lower than that in individuals with nonprotective haplotypes, indicating that polymorphisms in the *irf1* gene could be one of the determinants of HIV‐1 resistance [84]. Additionally, a study evaluating the early kinetics of the IRF1 response to IFN‐γ in the PBMCs of HESN patients and healthy controls revealed that both individuals exhibited a robust IRF1 response to exogenous IFN‐γ stimulation, but this response was rapidly “controlled” in HESN patients [85]. This could be mediated by low activation, as IRF1 seems to be highly expressed in activated cells [86]. In concordance, a study by Su et al. proposed that an IRF1 reduction in HESN can control the prolonged inflammatory state, allowing adequate induction of an antiviral response [87]. However, other authors have suggested that IRF1 may act as a key host cellular factor favoring HIV‐1 replication and early establishment of infection [88]. A study analyzing transcriptional profiles linked to HIV acquisition in a cohort from Africa revealed higher levels of the PTPRC gene, which is important for T‐cell activation and HIV‐1 infection, in seroconverter individuals before infection than in HESN individuals. There was also positive regulation of genes stimulated by IFN‐α and IFN‐γ in samples from seroconverter patients before infection compared with those from HESN individuals. This suggests the stimulation of regulatory responses; however, it can also lead to the recruitment of immune cells and the activation of target cells, increasing susceptibility to infection [89].

Finally, different proteins of the TIM family (T‐cell immunoglobulin and mucin‐like domains) are involved in immunological functions and are expressed in different cells of the immune system. TIM‐3 is expressed mainly on the surface of cytotoxic T lymphocytes, CD4^+^ T cells that secrete IFN‐γ, NK cells, NKT cells, monocytes, macrophages and dendritic cells (DCs), and it has been proposed to modulate the Th1 response [90, 91, 92]. Jones et al. reported that during progressive HIV‐1 infection, there is increased expression of Tim‐3 on CD8 + T cells that are specific to HIV‐1; additionally, these cells exhibit cytokine or proliferative impairment [93]. Interestingly, a polymorphism (rs4704846) in the HAVCR2 gene, which encodes the TIM‐3 protein, has been related to reduced susceptibility to HIV‐1 infection [94]. Moreover, the frequency of this polymorphism is significantly greater in HESN individuals than in HIV‐positive individuals, and it occurs independently of their route of exposure [94].

In the case of HIV‐1 infection, low activation also has some problems. HIV can establish a latent infection, remaining as a transcriptionally inactive provirus [95]; this latency is thought to contribute to the rebound of the viral load in the case of an antiviral treatment failure or interruption. Similarly, Treg cells that control immune activation can also contribute to the generation of these latent reservoirs; however, a possible solution has emerged: a therapy called “shock and kill” [96, 97]. This strategy eliminates viral reservoirs through the activation of viral replication through histone deacetylase inhibitors; thus, the immunological system eliminates the viral reservoir. However, current approaches cannot clear the virus, probably because of increased resistance to viral activation in cells with a low activation phenotype [98].

A low activation profile in HESN individuals may contribute to natural resistance to HIV‐1. This resistance is manifested by lower expression of activation markers and reduced production of pro‐inflammatory cytokines, suggesting a more effective immune response against the virus or reduced susceptibility to HIV infection. However, the persistence of HIV in a latent state and the challenge of overcoming resistance in low‐activation cells remain significant obstacles to treatment.

## 3 Activation Phenotype in HESN

Immune activation has been proposed as a potential protective mechanism against HIV‐1 infection. Unlike a low‐activation state, which may limit the immune system's response to infection, high immune activation could enhance the body's ability to counteract HIV‐1. This hypothesis is based on the idea that a robust immune response, characterized by increased expression of immune activation markers and cytokine production, might better equip the immune system to recognize and combat the virus. Specifically, immune activation can promote antiviral responses and facilitate the elimination of infected cells [99].

Despite evidence supporting the protective role of low activation in several cohorts of HESN individuals, the role of immune activation in HIV‐1 resistance has also been explored [100, 101, 102]. The increased expression of interferon‐stimulated genes (ISGs) and other immune‐related proteins, such as Myxovirus resistance 2 (MX2), complement system proteins (CR2, C4BPA), and endoplasmic reticulum aminopeptidase type 2 (ERAP 2), suggests that immune activation may also contribute to resistance by enhancing the antiviral capacity of the immune system [19].

Multiple mechanisms of activation have been explored; for example, TLR3, which recognizes double‐stranded RNA (dsRNA), can initiate innate immune reactions induced by pathogens such as HIV following the upregulation of NF‐kB and interferon regulatory factor 3 (IRF3), which results in elevated production of type I interferons and pro‐inflammatory cytokines, leading to an antiviral response [103]. Moreover, some studies have shown that the TLR‐3 rs3775291 C → T (Leu412Phe) T allele polymorphism is overrepresented in HIV‐1‐exposed seronegative intravenous drug users (HESN‐IDU) in comparison to HIV‐positive IDU; additionally, owing to TLR engagement, PBMCs isolated from subjects with this polymorphism exhibit greater resistance to ex vivo HIV‐1 infection and increased levels of pro‐inflammatory cytokines (IL‐1, IL‐6, IL‐10, IFN‐β, IFN‐γ, CCL3, RANTES, TNF‐α) and CD69 [104, 105]. However, this information contrasts with a critical study in which this polymorphism was associated with increased Coxsackie virus replication, leading to a greater risk of enteroviral myocarditis [106].

Other studies carried out in a HESN‐IDU cohort have shown a markedly immunologically activated phenotype, with lower percentages of naive CD8^+^ T cells and higher percentages of CD8^+^CD25^+^ and CD8^+^ CD38^+^ HLA‐DR^+^ T cells in HESN‐IDU than in healthy control individuals in a basal state [107]; the increased percentage of activated CD8^+^ T cells suggests a robust immune response that is ready to confront viral threats. These activated T cells are capable of recognizing and killing infected cells, which helps limit the spread of HIV‐1 [108]. Specifically, CD8^+^ T cells can release cytotoxic molecules such as perforin and granzymes that induce apoptosis in HIV‐infected cells [109]. Additionally, activated CD8^+^ T cells produce cytokines such as IFN‐γ, which enhances the antiviral response by inhibiting viral replication and recruiting other immune cells [110]. This heightened state of activation and functional capacity equips the immune system to counteract potential HIV‐1 infections more effectively. Although HIV‐1 can enter the bloodstream through various routes, the increased antiviral activity of these T cells provides a critical defense mechanism, potentially reducing the likelihood of the virus establishing a successful infection [111]. On the basis of this evidence, Tran and colleagues concluded that in HESN‐IDUs, cell activation seems closer to that of HIV‐1‐positive individuals than to that of negative controls, suggesting that the activation of immunological features does not necessarily favor HIV‐1 infection and, in contrast, could lead to natural resistance [107]. Additionally, in the same cohort, increased IFN‐γ and TNF‐α production by NK cells before and after stimulation with the K562 cell line compared with that in healthy controls and seroconverters was found in HESN‐IDUs [21].

The role of NK cells in natural resistance to HIV‐1 infection has been studied in depth, and an increase in NK cell activity related to an increase in cytotoxic capacity and the production of IFN‐γ, MIP1‐α, MIP‐1β, and RANTES has been described [112]. Interestingly, in a previous study conducted by us in MSM, we reported that individuals at high risk of HIV infection presented increased cytotoxic activity and a greater percentage of NK cells producing IFN‐γ than did low‐risk MSM controls [4]. Additionally, we observed an enhancement in the effector function of NK cells when they were cocultured with autologous CD4 + HIV + T cells, along with elevated levels of CD69 + IFN‐γ+ and CD69 + NKG2D + NK cells in high‐risk MSM compared with low‐risk MSM for HIV‐1 infection. NK cells exhibiting this phenotype play a crucial role in the immune response against HIV‐1 by actively participating in the recognition and elimination of infected cells. The upregulation of CD69, a marker associated with cellular activation and proliferation, indicates that these NK cells are in an activated state, which enhances their cytotoxic capabilities [113]. The presence of IFN‐γ, a key cytokine in antiviral responses, and NKG2D, an activating receptor that recognizes stress‐induced ligands on infected cells, further underscores their role in mounting a robust immune response [114, 115]. These findings suggest that NK cells with high levels of activation markers and effector functions contribute significantly to the control of HIV‐1 infection by targeting and killing HIV‐infected cells, thus playing a pivotal role in the natural resistance observed in high‐risk MSM populations. Table 1.

**Table 1:** Summary of Key Mechanisms in HIV‐1 Resistance Hypotheses. Treg cells, regulatory T cells; MIP‐1α/β, macrophage inflammatory protein 1 A/B; HLA‐DR, human leukocyte antigen–DR isotype; TLR, Toll‐like receptor; IRF1, Interferon Regulatory Factor 1; ISG, Interferon‐Stimulated Genes; MX2, Myxovirus resistance protein 2; IFN‐β/γ, Interferon β/γ; TNF‐α, Tumor Necrosis Factor α.

Summary of low immune activation mechanisms and associated molecules in HIV‐1 resistance		
Molecule	Mechanism	Reference
Treg cells	Regulation of immune response through cytokine production (e.g., IL‐10, IL‐35, TGF‐B1); control of cytotoxic T lymphocyte responses.	[55, 56, 57]
CD38, HLA‐DR, CD69	Contribute to HIV resistance by minimizing the availability of activated target cells for the virus to infect, thereby reducing the likelihood of viral replication and spread within the host.	[41, 59, 64]
MIP‐1α, MIP‐1β	Soluble factors that block HIV‐1 entry by binding to CCR5; higher production in HESN individuals.	[18, 65]
TLR expression	Reduced expression of TLRs in cervical mononuclear cells; appropriate antiviral responses after stimulation.	[68, 69]
IRF1	Transcription factor related to natural resistance; variations in expression affect response to IFN‐γ.	[70, 71, 72, 74, 76]
Summary of high immune activation mechanisms and associated molecules in HIV‐1 resistance		
TLR3	Recognizes specific PRRs, activates innate immune responses, upregulates NF‐kB and IRF3, resulting in elevated production of type I interferons and pro‐inflammatory cytokines.	[91]
CD8^+^CD25^+^ and CD8^+^CD38^+^ HLA^‐^DR^+^ T cells	Higher percentages in HESN‐IDU indicate a robust immune response, capable of recognizing and killing infected cells, and producing antiviral cytokines like IFN‐γ.	[95, 96, 97, 98]
NK Cells (CD69^+^, IFN^‐^γ^+^, NKG2D^+^)	Increased activation and effector capacity enhance cytotoxic capabilities, recognize and eliminate infected cells, produce cytokines like IFN‐γ, and express NKG2D receptor.	[100, 101, 102, 103]
ISGs (e.g., MX2)	Enhanced antiviral capacity through increased expression, contributing to resistance by improving immune responses against HIV‐1.	[11]
Cytokines (e.g., IL‐1, IL‐6, IL‐10, IFN‐β, IFN‐γ, TNF‐α)	Elevated levels associated with enhanced antiviral responses and increased resistance to HIV‐1 in certain cohorts.	[92, 93]
MIP‐1β	Interferes with the HIV infection process by blocking HIV‐1 entry through binding to CCR5, higher levels observed in HIV‐exposed uninfected children, suggesting a role in resistance.	[104]

**Figure 1:**
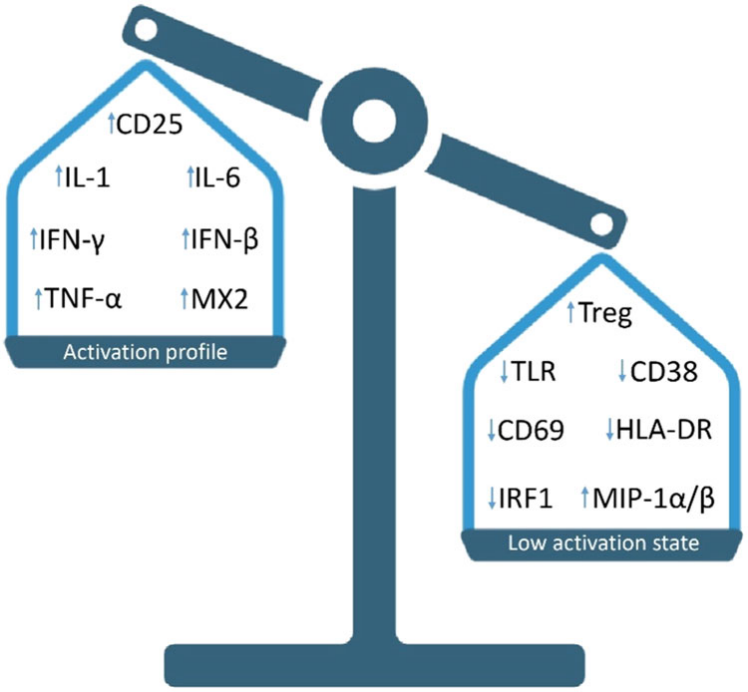
Schematic representation of the main mechanisms associated with activation and low‐activation phenotypes as correlates of protection against HIV infection in HESN individuals. Most of the available evidence suggests that resistance to infection is associated with a low activation phenotype in which a high frequency of Treg cells predominates; downregulation of activation markers, including CD25, CD39, CD69 and HLA‐DR; decreased levels of PRRs, such as TLR4/7/9; downregulation of the transcription factor IRF‐1; and increased levels of MIP‐1α/β. However, other authors describe an activation phenotype as a mechanism of protection against infection, characterized by elevated expression of activation markers, including CD2; high levels of ISGs, such as MX2; and pro‐inflammatory cytokines, such as IL‐1/6, IFN‐β/γ and TNF‐α.

Additionally, a study of HIV‐exposed uninfected children born to HIV‐1‐infected women in the city of Sao Paulo, Brazil, revealed that these children had a greater percentage of CD38^+^ and HLA‐DR^+^ CD8^+^ T cells at 12 months of age and a greater percentage of CD38^+^HLA^‐^DR^+^CD4^+^ T cells at 6–12 years of age than did uninfected controls of the same age. Furthermore, the levels of plasma cytokines such as IL‐2, IL‐6, IL‐7, IL‐10, IL‐12p70, IL‐13, IL‐17, IFN‐γ, TNF‐α, G‐CSF, GM‐CSF and MCP‐1 were similar; an increase was detected at 12 months, but lower levels of these cytokines were detected at 6–12 years of age in both groups [116]. In addition, the plasma level of MIP‐1β, a natural ligand for CCR5 that may interfere with the HIV infection process, was greater in HIV‐exposed uninfected children at birth than in uninfected controls [116]. This evidence suggests that immune activation may be partially involved in HIV‐1 resistance. Figure 1.

Studies on HESN show an apparent contradiction regarding the contribution of immune activation to natural resistance against the virus. This discrepancy may be due to several factors: (i) the characteristics of the studied populations, such as age, sex, genetics, and frequency of exposure to the virus, which can affect the immune response. Age, level of exposure to HIV‐1, and genetic variants in individuals are key elements in these differences. In fact, HESN individuals do not constitute a homogeneous group; some may have been repeatedly exposed to the virus, whereas others have been exposed less frequently. These variations in exposure may result in different levels of immune activation compared with other cohorts with less exposure or different demographic characteristics. (ii) Experimental design: levels of immune activation vary depending on the time elapsed since exposure to the virus. Studies that collect samples early may capture an acute and rapid response (high activation), whereas studies that take samples later might observe a more stable immunity related to low activation. (iii) Methods used to assess activation: Techniques such as flow cytometry or cytokine profiling analyze different components of the immune response. For example, flow cytometry can detect specific activation markers on T cells, while cytokine profiles reveal a broader pro‐inflammatory landscape. Depending on the technique, the results may indicate either high or low activation. (iv) Geographical and environmental factors: exposure to other pathogens (bacteria, parasites) and environmental conditions can influence baseline levels of immune activation. In areas with high exposure to pathogens, baseline activation levels tend to be relatively high, which may complicate interpretations of whether high or low activation is protective against HIV. (v) Definition of immune activation: different studies use varying criteria to define activation, whether on the basis of cytokine production, expression of activation markers, or cell proliferation. These differences in definition can lead to divergent interpretations of whether a more active and rapid immune response or, conversely, a more moderate and controlled response is what provides protection against infection.

Therefore, it is essential to consider the context of each study, including the characteristics of the participants and the type of HIV exposure, to better understand how these factors affect immune activation and its role in virus resistance. The reconciliation of these seemingly contradictory data may be due to the use of a more nuanced approach that considers the diversity of immune responses among HESN individuals and the specific conditions under which these responses occur.

Moreover, it is important to note that although we address these two phenomena independently in this analysis, they likely co‐occur and play complementary roles in individuals. In this sense, within the same individual, a global reduction in the activation of specific immune factors (e.g., T cells in mucosal tissues) could be observed to limit the number of cells susceptible to the virus. In contrast, other specific components (such as NK cells or CD8^+^ T cells) might be selectively activated to respond effectively to the infection. Additionally, we consider it feasible that these mechanisms act sequentially, depending on the time elapsed since exposure to HIV. Initially, selective activation of specific immune cells could be crucial for containing the infection. In contrast, a low activation phenotype at later stages could help minimize chronic inflammation and prevent viral spread.

## 4 Activation and Low‐Activation Phenotypes Associated With the Control of HIV‐1 Infection

The following is an additional section that examines the mechanisms associated with HIV‐1 suppression in individuals who naturally control the infection. This study seeks to offer a complementary perspective that highlights the complexity of HIV‐1 resistance, thereby enriching the overall understanding of low‐ and high‐activation phenomena.

Individuals with HIV‐1 infection have been categorized into distinct groups on the basis of their ability to manage the virus. Elite controllers (ECs) are those who spontaneously suppress viremia to undetectable levels (< 50 copies/mL) and maintain high CD4^+^ counts (200–1000/µL) without antiretroviral therapy. Viremic controllers (VCs) can suppress the viral load to less than 2000 copies/mL and maintain a healthy CD4^+^ T‐cell count, whereas noncontrollers, who are receiving antiretroviral therapy, achieve low viral loads under treatment [117, 118].

In ECs, evidence suggests that CD8^+^ T cells, particularly central memory cells, exhibit greater activation and functional capacity—such as cytokine production, proliferation, and cytotoxicity—than VSs do, contributing to effective viral control without progression to AIDS [119, 120]. In this context, some reports have suggested that higher levels of CD8 + T‐cell activation in ECs might be related to higher levels of HIV‐specific CD8^+^ T cells [121], low levels of Treg cells [122], microbial translocation phenomena [123] and coinfections with other viral agents, such as cytomegalovirus, Epstein‒Barr virus and hepatitis C virus [124].

The systemic cytokine response in ECs has also been linked to their ability to control HIV replication. Specifically, studies have shown that these individuals exhibit elevated levels of cytokines such as SDF‐1, CCL14, CCL21, CCL27, and XCL1 compared with noncontrollers. This combination of cytokines seems to exert an antiviral effect by suppressing the replication of the R5 and X4 strains of the virus in resting T cells. Additionally, these cytokines modulate the expression of key receptors on CD4 + T cells, increasing the levels of CD69 and CCR5 while reducing the expression of CXCR4 and CCR7. This regulation may be crucial in limiting viral entry and spread in these cells [125]. Additionally, increased expression of mTOR and eIF2, a family of molecules involved in signal transduction related to crucial cellular functions, including the regulation of growth, metabolism, cell proliferation, transcriptional control, and latency in the main target of infection, CD4^+^ T cells, has been reported [126].

However, other reports indicate that infection control is associated with a state of low activation. For example, in an analysis conducted in ECs using purified CD4^+^ and CD8^+^ T cells, a significant decrease in T‐cell activation was observed compared with that in cells from HIV^+^ patients receiving antiretroviral therapy. This study also revealed the downregulation of genes associated with the inflammatory response, including pathways such as Toll‐like receptors (TLRs) and TREM1, as well as pro‐inflammatory genes such as IL‐8/CXCL8, CXCR1, and CXCR2 [127, 128]. These cytokines are linked to persistent inflammation and the development of disease in individuals infected with HIV, suggesting that their reduction in ECs may be related to effective viral control. Additionally, ECs presented higher levels of HIV‐specific IL‐2^+^ IFN‐γ^+^ T cells and decreased expression of the activation marker CD38 than noncontrollers did [129]. In this group, the HIV‐1 restriction factor X‐DING‐CD4, which is associated with the transcriptional blockade of the viral LTR and the pro‐inflammatory response, is also elevated in ECs compared with noncontrollers and HIV‐negative individuals [130, 131]. These findings highlight the importance of precise immunological regulation and innate resistance in the natural control of HIV, providing deeper insights into the underlying mechanisms that allow ECs to maintain an undetectable viral load and controlled inflammation.

Moreover, through the study of the transcriptional profiles of CD4^+^ and CD8^+^ T cells in ECs, it has been shown that the phenotype of HIV infection control is associated with differential expression of proteins such as KZNF, which is implicated in the repression of regulatory elements derived from transposable elements for genes related to antiviral activity, leading to increased resistance to HIV infection [132]. From this perspective, the control of HIV‐1 replication in ECs is associated with low expression of activation receptors and decreased transcription of genes involved in the inflammatory response and the regulation of cellular function.

## 5 Conclusion

Research on HESN individuals has identified natural resistance mechanisms to HIV‐1 that extend beyond the well‐known CCR5Δ32 mutation, involving both innate and adaptive immune reactions, including T‐cell activation and the function of NK cells. While a low‐activation phenotype, characterized by reduced expression of markers such as CD25, CD38, and HLA‐DR, along with lower production of pro‐inflammatory cytokines in peripheral and mucosal tissues, appears to play a key role in reducing the number of activated cells susceptible to infection, some studies suggest that immune activation might also act as a protective mechanism. This low‐activation phenotype not only limits viral replication but also minimizes chronic inflammation, which would otherwise facilitate viral entry and spread. The complexity of these immune responses, reflected in the conflicting evidence concerning whether low or high immune activation offers protection against infection, suggests that there may be multiple pathways to HIV‐1 resistance, influenced by factors such as the type of viral exposure, the immune environment, and individual genetics. This underscores the need for further research to determine which immune states are truly protective and how different activation mechanisms interact with one another. As these differences are explored, it will be essential to better understand how these responses can be modulated, not only to prevent infection but also to develop more effective treatments that leverage these particular immune states. Future research should focus on clarifying these aspects, evaluating how immune activation and low activation interact in HESN individuals, and translating these insights into improved clinical interventions.

## Author Contributions

**María M Naranjo‐Covo:** investigation, writing–original draft, writing–review and editing. **Daniel S. Rincón‐Tabares:** investigation, writing–original draft, writing–review and editing. **Lizdany Flórez‐Álvarez:** writing–review and editing. **Juan C. Hernandez:** supervision, writing–review and editing. **Wildeman Zapata‐Builes:** supervision, writing–original draft, writing–review and editing.

## References

[1] M. Miyazawa , L. Lopalco , F. Mazzotta , et al., “The Immunologic Advantage of HIV‐Exposed Seronegative Individuals,” AIDS 23, no. 2 (2009): 161–175. 19098485 10.1097/QAD.0b013e3283196a80

[2] K. R. Fowke , N. J. Nagelkerke , J. Kimani , et al., “Resistance to HIV‐1 Infection Among Persistently Seronegative Prostitutes in Nairobi, Kenya,” Lancet 348, no. 9038 (1996): 1347–1351. 8918278 10.1016/S0140-6736(95)12269-2

[3] R. E. Horton , P. J. McLaren , K. Fowke , J. Kimani , and T. B. Ball , “Cohorts for the Study of HIV‐1‐Exposed but Uninfected Individuals: Benefits and Limitations,” Journal of Infectious Diseases 202, no. Suppl 3 (2010): S377–S381. 20887228 10.1086/655971

[4] L. Flórez‐Álvarez , Y. Blanquiceth , K. Ramírez , et al., NK Cell Activity and CD57+/NKG2Chigh Phenotype Are Increased in Men Who Have Sex With Men at High Risk for HIV. Front Immunol [Internet]. 2020 [citado 6 de septiembre de 2023];11. Disponible en: https://www.frontiersin.org/articles/10.3389/fimmu.2020.537044. 10.3389/fimmu.2020.537044 PMC7517039 33042136

[5] N. Taborda , W. Zapata‐Builes , C. Montoya , and M. T. Rugeles , “Short Communication: Increased Expression of Secretory Leukocyte Protease Inhibitor in Oral Mucosa of Colombian HIV Type 1‐Exposed Seronegative Individuals,” AIDS Research and Human Retroviruses 28, no. 9 (2012): 1059–1062. 22149181 10.1089/aid.2011.0151 PMC3423646

[6] C. J. Montoya , P. A. Velilla , C. Chougnet , A. L. Landay , and M. T. Rugeles , “Increased IFN‐γ Production by NK and CD3+/CD56+ Cells in Sexually HIV‐1‐Exposed but Uninfected Individuals,” Clinical Immunology 120, no. 2 (2006): 138–146. 16624619 10.1016/j.clim.2006.02.008

[7] A. Gupta and H. Padh , “The Global Distribution of CCR5 Delta 32 Polymorphism: Role in HIV‐1 Protection,” BMC Infectious Diseases 12, no. Suppl 1 (2012): O16.

[8] D. H. Jang , B. S. Choi , and S. S. Kim , “The Effects of RANTES/CCR5 Promoter Polymorphisms on HIV Disease Progression in HIV‐Infected Koreans,” International Journal of Immunogenetics 35, no. 2 (2008): 101–105. 18218038 10.1111/j.1744-313X.2007.00743.x

[9] rs333 (INDEL) ‐ Population genetics ‐ Homo_sapiens ‐ Ensembl genome browser 111 [Internet]. [citado 24 de abril de 2024]. Disponible en: https://www.ensembl.org/Homo_sapiens/Variation/Population?db=core;r=3:46372953-46373987;v=rs333;vdb=variation;vf=183253707.

[10] S. Alarcón‐Uribe , W. Zapata‐Builes , and L. F. Higuita‐Gutiérrez , “Resistencia Natural a La Infección Por El VIH‐1. Revisión Sistemática De La Literatura,” Iatreia 37, no. 1 (2023): 63–84, https://revistas.udea.edu.co/index.php/iatreia/article/view/349513.

[11] P. J. R. Goulder and B. D. Walker , “HIV and HLA Class I: An Evolving Relationship,” Immunity 37, no. 3 (2012): 426–440. 22999948 10.1016/j.immuni.2012.09.005 PMC3966573

[12] D. van Manen, , A. B. van Wout, , and H. Schuitemaker , “Genome‐Wide Association Studies on HIV Susceptibility, Pathogenesis and Pharmacogenomics,” Retrovirology 9 (2012): 70. 22920050 10.1186/1742-4690-9-70 PMC3468375

[13] D. Zipeto and A. Beretta , “HLA‐C and HIV‐1: Friends or Foes?,” Retrovirology 9, no. 1 (2012): 39. 22571741 10.1186/1742-4690-9-39 PMC3386009

[14] H. Do , A. Vasilescu , G. Diop , et al., “Exhaustive Genotyping of the CEM15 (APOBEC3G) Gene and Absence of Association With AIDS Progression in a French Cohort,” Journal of Infectious Diseases 191, no. 2 (2005): 159–163. 15609224 10.1086/426826

[15] APOBEC3G Genetic Variants and Their Influence on the Progression to AIDS | Journal of Virology [Internet]. [citado 8 de diciembre de 2024]. Disponible en: 10.1128/jvi.78.20.11070-11076.2004. PMC521814 15452227

[16] K. Reddy , C. A. Winkler , L. Werner , et al, “APOBEC3G Expression is Dysregulated in Primary HIV‐1 Infection and Polymorphic Variants Influence CD4+ T‐Cell Counts and Plasma Viral Load,” AIDS 24, no. 2 (2010): 195–204. 19996938 10.1097/QAD.0b013e3283353bba PMC3470914

[17] H. Javanbakht , P. An , B. Gold , et al., “Effects of Human TRIM5α Polymorphisms on Antiretroviral Function and Susceptibility to Human Immunodeficiency Virus Infection,” Virology 354, no. 1 (2006): 15–27. 16887163 10.1016/j.virol.2006.06.031

[18] Polymorphisms of the SAMHD1 Gene Are Not Associated With the Infection and Natural Control of HIV Type 1 in Europeans and African‐Americans. ResearchGate [Internet]. 22 de octubre de 2024 [citado 8 de diciembre de 2024]; Disponible en: https://www.researchgate.net/publication/224834546_Polymorphisms_of_the_SAMHD1_Gene_Are_Not_Associated_with_the_Infection_and_Natural_Control_of_HIV_Type_1_in_Europeans_and_African-Americans. 10.1089/aid.2012.0039 PMC3505062 22530776

[19] C. Fenizia , J. F. Rossignol , M. Clerici , and M. Biasin , “Genetic and Immune Determinants of Immune Activation in HIV‐Exposed Seronegative Individuals and Their Role in Protection Against HIV Infection,” Infection, Genetics and Evolution: Journal of Molecular Epidemiology and Evolutionary Genetics in Infectious Diseases 66 (2018): 325–334. 29258786 10.1016/j.meegid.2017.12.014

[20] E. S. Rosenberg , J. M. Billingsley , A. M. Caliendo , et al., “Vigorous HIV‐1‐Specific CD4+ T Cell Responses Associated With Control of Viremia,” Science 278, no. 5342 (1997): 1447–1450. 9367954 10.1126/science.278.5342.1447

[21] D. Scott‐Algara , L. X. Truong , P. Versmisse , et al., “Cutting Edge: Increased NK Cell Activity in HIV‐1‐Exposed But Uninfected Vietnamese Intravascular Drug Users,” Journal of Immunology 171, no. 11 (2003): 5663–5667. 10.4049/jimmunol.171.11.5663 14634071

[22] L. Sun , C. M. Finnegan , T. Kish‐Catalone , et al., “Human β‐Defensins Suppress Human Immunodeficiency Virus Infection: Potential Role in Mucosal Protection,” Journal of Virology 79, no. 22 (2005): 14318–14329. 16254366 10.1128/JVI.79.22.14318-14329.2005 PMC1280242

[23] W. Zapata , B. Rodriguez , J. Weber , et al., “Increased Levels of Human Beta‐Defensins mRNA in Sexually HIV‐1 Exposed but Uninfected Individuals,” Current HIV Research 6, no. 6 (2008): 531–538. 18991618 10.2174/157016208786501463 PMC4126611

[24] F. A. Koning , C. A. Jansen , J. Dekker , et al., “Correlates of Resistance to HIV‐1 Infection in Homosexual Men With High‐Risk Sexual Behaviour,” AIDS 18, no. 8 (2004): 1117–1126. 15166527 10.1097/00002030-200405210-00005

[25] S. M. Iqbal , T. B. Ball , P. Levinson , et al., “Elevated Elafin/Trappin‐2 in the Female Genital Tract is Associated With Protection Against HIV Acquisition,” AIDS 23, no. 13 (2009): 1669–1677. 19553806 10.1097/QAD.0b013e32832ea643

[26] A. Burgener , S. Rahman , R. Ahmad , et al., “Comprehensive Proteomic Study Identifies Serpin and Cystatin Antiproteases as Novel Correlates of HIV‐1 Resistance in the Cervicovaginal Mucosa of Female Sex Workers,” Journal of Proteome Research 10, no. 11 (2011): 5139–5149. 21973077 10.1021/pr200596r

[27] S. M. Gonzalez , N. A. Taborda , M. G. Feria , et al., “High Expression of Antiviral Proteins in Mucosa From Individuals Exhibiting Resistance to Human Immunodeficiency Virus,” PLoS One 10, no. 6 (2015): e0131139. 26091527 10.1371/journal.pone.0131139 PMC4474690

[28] C. Devito , K. Broliden , R. Kaul , et al., “Mucosal and Plasma IgA From HIV‐1‐Exposed Uninfected Individuals Inhibit HIV‐1 Transcytosis Across Human Epithelial Cells,” Journal of Immunology 165, no. 9 (2000): 5170–5176. 10.4049/jimmunol.165.9.5170 11046049

[29] T. Hirbod , R. Kaul , C. Reichard , et al., “HIV‐Neutralizing Immunoglobulin A and HIV‐Specific Proliferation are Independently Associated With Reduced HIV Acquisition in Kenyan Sex Workers,” AIDS 22, no. 6 (2008): 727–735. 18356602 10.1097/QAD.0b013e3282f56b64

[30] P. A. Serna‐Ortega , W. Aguilar‐Jimenez , L. Florez‐Álvarez , D. Trabattoni , M. T. Rugeles , and M. Biasin , “IL‐21 Is Associated With Natural Resistance to HIV‐1 Infection in a Colombian HIV Exposed Seronegative Cohort,” Microbes and Infection 22, no. 8 (2020): 371–374. 31816393 10.1016/j.micinf.2019.11.002

[31] P. A. S. Ortega , I. Saulle , V. Mercurio , et al., “Interleukin 21 (IL‐21)/microRNA‐29 (miR‐29) Axis is Associated With Natural Resistance to HIV‐1 Infection,” AIDS 32, no. 17 (2018): 2453–2461. 30005016 10.1097/QAD.0000000000001938

[32] J. R. Salkowitz , S. F. Purvis , H. Meyerson , et al., “Characterization of High‐Risk HIV‐1 Seronegative Hemophiliacs,” Clinical Immunology 98, no. 2 (2001): 200–211. 11161976 10.1006/clim.2000.4969

[33] F. A. Koning , S. A. Otto , M. D. Hazenberg , et al., “Low‐Level CD4+ T Cell Activation is Associated With Low Susceptibility to HIV‐1 Infection,” Journal of Immunology 175, no. 9 (2005): 6117–6122. 10.4049/jimmunol.175.9.6117 16237108

[34] P. J. McLaren , T. B. Ball , C. Wachihi , et al., “HIV‐Exposed Seronegative Commercial Sex Workers Show a Quiescent Phenotype in the CD4+ T Cell Compartment and Reduced Expression of HIV‐Dependent Host Factors,” Journal of Infectious Diseases 202, no. Suppl 3 (2010): S339–S344. 20887221 10.1086/655968

[35] M. Biasin , M. Clerici , and L. Piacentini , “Innate Immunity in Resistance to Hiv Infection,” Journal of Infectious Diseases 202, no. Suppl 3 (2010): S361–S365. 20887225 10.1086/655965

[36] L. Zhang , W. Yu , T. He , et al., “Contribution of Human α‐Defensin 1, 2, and 3 to the Anti‐HIV‐1 Activity of CD8 Antiviral Factor,” Science 298, no. 5595 (2002): 995–1000. 12351674 10.1126/science.1076185

[37] L. Furci , G. Scarlatti , S. Burastero , et al., “Antigen‐Driven C‐C Chemokine‐Mediated HIV‐1 Suppression By CD4(+) T Cells From Exposed Uninfected Individuals Expressing the Wild‐Type CCR‐5 Allele,” Journal of Experimental Medicine 186, no. 3 (1997): 455–460. 9236198 10.1084/jem.186.3.455 PMC2198997

[38] N. M. Chapman and H. Chi , “Hallmarks of T‐Cell Exit From Quiescence,” Cancer Immunology Research 6, no. 5 (2018): 502–508. 29716982 10.1158/2326-6066.CIR-17-0605

[39] T. Oki , K. Nishimura , J. Kitaura , et al., “A Novel Cell‐Cycle‐Indicator, mVenus‐p27K−, Identifies Quiescent Cells and Visualizes G0–G1 Transition,” Scientific Reports 4, no. 1 (2014): 4012. 24500246 10.1038/srep04012 PMC3915272

[40] N. Urbán and T. H. Cheung , “Stem Cell Quiescence: The Challenging Path to Activation,” Development 148, no. 3 (2021): dev165084. 33558315 10.1242/dev.165084 PMC7888710

[41] V. Gire and V. Dulić , “Senescence from G2 Arrest, Revisited,” Cell Cycle 14, no. 3 (2015): 297–304. 25564883 10.1080/15384101.2014.1000134 PMC4353294

[42] A. de Morree and T. A. Rando , “Regulation of Adult Stem Cell Quiescence and its Functions in the Maintenance of Tissue Integrity,” Nature Reviews Molecular Cell Biology 24, no. 5 (2023): 334–354. 36922629 10.1038/s41580-022-00568-6 PMC10725182

[43] M. Mitra , L. D. Ho , and H. A. Coller , “An In Vitro Model of Cellular Quiescence in Primary Human Dermal Fibroblasts,” Methods in Molecular Biology 1686 (2018): 27–47. 29030810 10.1007/978-1-4939-7371-2_2 PMC5718883

[44] S. S. Hwang , J. Lim , Z. Yu , et al., “mRNA Destabilization by BTG1 and BTG2 Maintains T Cell Quiescence,” Science 367, no. 6483 (2020): 1255–1260. 32165587 10.1126/science.aax0194

[45] D. N. Vatakis , C. C. Nixon , and J. A. Zack Quiescent T Cells and HIV: An Unresolved Relationship. Immunol Res [Internet]. diciembre de 2010 [citado 23 de abril de 2024];48(0). Disponible en: https://www.ncbi.nlm.nih.gov/pmc/articles/PMC3824961/. 10.1007/s12026-010-8171-0 PMC3824961 20725862

[46] J. A. Zack , A. M. Haislip , P. Krogstad , and I. S. Chen , “Incompletely Reverse‐Transcribed Human Immunodeficiency Virus Type 1 Genomes in Quiescent Cells can Function as Intermediates in the Retroviral Life Cycle,” Journal of Virology 66, no. 3 (1992): 1717–1725. 1371173 10.1128/jvi.66.3.1717-1725.1992 PMC240919

[47] H. Zhou , M. Xu , Q. Huang , et al., “Genome‐Scale Rnai Screen for Host Factors Required for HIV Replication,” Cell Host & Microbe 4, no. 5 (2008): 495–504. 18976975 10.1016/j.chom.2008.10.004

[48] R. König , Y. Zhou , D. Elleder , et al., “Global Analysis of Host‐Pathogen Interactions That Regulate Early Stage HIV‐1 Replication,” Cell 135, no. 1 (2008): 49–60. 18854154 10.1016/j.cell.2008.07.032 PMC2628946

[49] B. Roche , B. Arcangioli , and R. Martienssen , “Transcriptional Reprogramming in Cellular Quiescence,” RNA Biology 14, no. 7 (2017): 843–853. 28497998 10.1080/15476286.2017.1327510 PMC5546717

[50] M. Röhl , A. Tjernlund , J. Lajoie , et al., “HIV‐Exposed Seronegative Sex Workers Express Low T‐Cell Activation and an Intact Ectocervical Tissue Microenvironment,” Vaccines 9, no. 3 (2021): 217. 33806390 10.3390/vaccines9030217 PMC7998094

[51] R. Valdor and F. Macian , “Induction and Stability of the Anergic Phenotype in T Cells,” Seminars in Immunology 25, no. 4 (2013): 313–320. 24211041 10.1016/j.smim.2013.10.010 PMC3927995

[52] C. T. Kuo , M. L. Veselits , and J. M. Leiden , “LKLF: A Transcriptional Regulator of Single‐Positive T Cell Quiescence and Survival,” Science 277, no. 5334 (1997): 1986–1990. 9302292 10.1126/science.277.5334.1986

[53] D. Tzachanis , G. J. Freeman , N. Hirano , et al., “Tob Is a Negative Regulator of Activation That Is Expressed in Anergic and Quiescent T Cells,” Nature Immunology 2, no. 12 (2001): 1174–1182. 11694881 10.1038/ni730

[54] P. J. Coffer , “Transcriptional Regulation of Lymphocyte Quiescence: As Cunning as a FOX,” Trends in Immunology 24, no. 9 (2003): 470–471. 12967666 10.1016/s1471-4906(03)00205-9

[55] R. H. Schwartz , “A Cell Culture Model for T Lymphocyte Clonal Anergy,” Science 248, no. 4961 (1990): 1349–1356. 2113314 10.1126/science.2113314

[56] J. Crespo , H. Sun , T. H. Welling , Z. Tian , and W. Zou , “T Cell Anergy, Exhaustion, Senescence, and Stemness in the Tumor Microenvironment,” Current Opinion in Immunology 25, no. 2 (2013): 214–221. 23298609 10.1016/j.coi.2012.12.003 PMC3636159

[57] Y. D. Korin and J. A. Zack , “Progression to the G1b Phase of the Cell Cycle is Required for Completion of Human Immunodeficiency Virus Type 1 Reverse Transcription in T Cells,” Journal of Virology 72, no. 4 (1998): 3161–3168. 9525642 10.1128/jvi.72.4.3161-3168.1998 PMC109773

[58] Z. Darzynkiewicz , T. Sharpless , L. Staiano‐Coico , and M. R. Melamed , “Subcompartments of the G1 Phase of Cell Cycle Detected by Flow Cytometry,” Proceedings of the National Academy of Sciences 77, no. 11 (1980): 6696–6699. 10.1073/pnas.77.11.6696 PMC350355 6161370

[59] J. Bullwinkel , B. Baron‐Lühr , A. Lüdemann , C. Wohlenberg , J. Gerdes , and T. Scholzen , “Ki‐67 Protein is Associated With Ribosomal RNA Transcription in Quiescent and Proliferating Cells,” Journal of Cellular Physiology 206, no. 3 (2006): 624–635. 16206250 10.1002/jcp.20494

[60] I. Miller , M. Min , C. Yang , et al., “Ki67 is a Graded Rather Than a Binary Marker of Proliferation Versus Quiescence,” Cell Reports 24, no. 5 (2018): 1105–1112.e5. 30067968 10.1016/j.celrep.2018.06.110 PMC6108547

[61] S. A. Ezhevsky , A. Ho , M. Becker‐Hapak , P. K. Davis , and S. F. Dowdy , “Differential Regulation of Retinoblastoma Tumor Suppressor Protein by G(1) Cyclin‐Dependent Kinase Complexes In Vivo,” Molecular and Cellular Biology 21, no. 14 (2001): 4773–4784. 11416152 10.1128/MCB.21.14.4773-4784.2001 PMC87164

[62] C. M. Card , T. B. Ball , and K. R. Fowke , “Immune Quiescence: A Model of Protection Against HIV Infection,” Retrovirology 10 (2013): 141. 24257114 10.1186/1742-4690-10-141 PMC3874678

[63] L. Strauss , C. Bergmann , M. Szczepanski , W. Gooding , J. T. Johnson , and T. L. Whiteside , “A Unique Subset of CD4+CD25highFoxp3+ T Cells Secreting Interleukin‐10 and Transforming Growth Factor‐β1 Mediates Suppression in the Tumor Microenvironment,” Clinical Cancer Research 13, no. 15 Pt 1 (2007): 4345–4354. 17671115 10.1158/1078-0432.CCR-07-0472

[64] L. W. Collison , C. J. Workman , T. T. Kuo , et al., “The Inhibitory Cytokine IL‐35 Contributes to Regulatory T‐Cell Function,” Nature 450, no. 7169 (2007): 566–569. 18033300 10.1038/nature06306

[65] M. Ahmadzadeh and S. A. Rosenberg , “TGF‐Beta 1 Attenuates the Acquisition and Expression of Effector Function by Tumor Antigen‐Specific Human Memory CD8 T Cells,” Journal of Immunology 174, no. 9 (2005): 5215–5223. 10.4049/jimmunol.174.9.5215 PMC2562293 15843517

[66] J. D. Estes , Q. Li , M. R. Reynolds , et al., “Premature Induction of an Immunosuppressive Regulatory T Cell Response during Acute Simian Immunodeficiency Virus Infection,” Journal of Infectious Diseases 193, no. 5 (2006): 703–712. 16453267 10.1086/500368

[67] C. M. Card , P. J. McLaren , C. Wachihi , J. Kimani , F. A. Plummer , and K. R. Fowke , “Decreased Immune Activation in Resistance to HIV‐1 Infection Is Associated With an Elevated Frequency of CD4(+)CD25(+)FOXP3(+) Regulatory T Cells,” Journal of Infectious Diseases 199, no. 9 (2009): 1318–1322. 19301980 10.1086/597801

[68] R. Shen , H. E. Richter , and P. D. Smith , “Interactions Between HIV‐1 and Mucosal Cells in the Female Reproductive Tract,” American Journal of Reproductive Immunology 71, no. 6 (2014): 608–617. 24689653 10.1111/aji.12244 PMC4073589

[69] P. Patel , C. B. Borkowf , J. T. Brooks , A. Lasry , A. Lansky , and J. Mermin , “Estimating Per‐Act HIV Transmission Risk: A Systematic Review,” AIDS 28, no. 10 (2014): 1509–1519. 24809629 10.1097/QAD.0000000000000298 PMC6195215

[70] E. Mykhalovskiy , J. G. Betteridge , and D. McLay HIV Non‐Disclosure and the Criminal Law: Establishing Policy Options for Ontario. SSRN Electron J [Internet]. 2010 [citado 29 de abril de 2024]; Disponible en: http://www.ssrn.com/abstract=1747844.

[71] M. C. Boily , R. F. Baggaley , L. Wang , et al., “Heterosexual Risk of HIV‐1 Infection Per Sexual Act: Systematic Review and Meta‐Analysis of Observational Studies,” Lancet Infectious Diseases 9, no. 2 (2009): 118–129. 19179227 10.1016/S1473-3099(09)70021-0 PMC4467783

[72] D. A. Walsh , H. Borges da Silva , L. K. Beura , et al., “The Functional Requirement for CD69 in Establishment of Resident Memory CD8+ T Cells Varies With Tissue Location,” Journal of Immunology 203, no. 4 (2019): 946–955. 10.4049/jimmunol.1900052 PMC6684481 31243092

[73] L. Kestens , G. Vanham , C. Vereecken , et al., “Selective Increase of Activation Antigens Hla‐Dr and CD38 on CD45RO+ T Lymphocytes During HIV‐1 Infection,” Clinical and Experimental Immunology 95, no. 3 (1994): 436–441. 7907956 10.1111/j.1365-2249.1994.tb07015.x PMC1535073

[74] S. M. Gonzalez , N. A. Taborda , L. A. Correa , et al., “Particular Activation Phenotype of T Cells Expressing Hla‐Dr but Not CD38 in GALT From HIV‐Controllers is Associated With Immune Regulation and Delayed Progression to AIDS,” Immunologic Research 64, no. 3 (2016): 765–774. 26724942 10.1007/s12026-015-8775-5

[75] A. L. DeVico and R. C. Gallo , “Control of HIV‐1 Infection by Soluble Factors of the Immune Response,” Nature Reviews Microbiology 2, no. 5 (2004): 401–413. 15100693 10.1038/nrmicro878

[76] G. Ma , T. Greenwell‐Wild , K. Lei , et al., “Secretory Leukocyte Protease Inhibitor Binds to Annexin II, a Cofactor for Macrophage HIV‐1 Infection,” The Journal of Experimental Medicine 200, no. 10 (2004): 1337–1346. 15545357 10.1084/jem.20041115 PMC2211913

[77] J. Lajoie , J. Juno , A. Burgener , et al., “A Distinct Cytokine and Chemokine Profile at the Genital Mucosa is Associated With HIV‐1 Protection Among HIV‐Exposed Seronegative Commercial Sex Workers,” Mucosal Immunology 5, no. 3 (2012): 277–287. 22318497 10.1038/mi.2012.7

[78] J. A. Fulcher , L. Romas , J. C. Hoffman , et al., “Highly Human Immunodeficiency Virus‐Exposed Seronegative Men Have Lower Mucosal Innate Immune Reactivity,” AIDS Research and Human Retroviruses 33, no. 8 (2017): 788–795. 28503933 10.1089/aid.2017.0014 PMC5564013

[79] X. D. Yao , R. W. Omange , B. M. Henrick , et al., “Acting Locally: Innate Mucosal Immunity in Resistance to HIV‐1 Infection in Kenyan Commercial Sex Workers,” Mucosal Immunology 7, no. 2 (2014): 268–279. 23801306 10.1038/mi.2013.44

[80] B. M. Henrick , X. D. Yao , M. A. Zahoor , A. Abimiku , S. Osawe , and K. L. Rosenthal , “TLR10 Senses HIV‐1 Proteins and Significantly Enhances HIV‐1 Infection,” Frontiers in Immunology 10 (2019): 482. 30930906 10.3389/fimmu.2019.00482 PMC6430187

[81] T. Taniguchi , K. Ogasawara , A. Takaoka , and N. Tanaka , “IRF Family of Transcription Factors as Regulators of Host Defense,” Annual Review of Immunology 19 (2001): 623–655. 10.1146/annurev.immunol.19.1.623 11244049

[82] T. B. Ball , H. Ji , J. Kimani , et al., “Polymorphisms in IRF‐1 Associated With Resistance to HIV‐1 Infection in Highly Exposed Uninfected Kenyan Sex Workers,” AIDS 21, no. 9 (2007): 1091–1101. 17502719 10.1097/QAD.0b013e3280ef6ae1

[83] A. Sivro , L. R. McKinnon , H. Ji , et al., “Interferon Regulatory Factor 1 Polymorphisms Previously Associated With Reduced HIV Susceptibility Have no Effect on HIV Disease Progression,” PLoS One 8, no. 6 (2013): e66253. 23799084 10.1371/journal.pone.0066253 PMC3683001

[84] H. Ji , T. B. Ball , Z. Ao , J. Kimani , X. Yao , and F. A. Plummer , “Reduced HIV‐1 Long Terminal Repeat Transcription in Subjects With Protective Interferon Regulatory Factor‐1 Genotype: A Potential Mechanism Mediating Resistance to Infection by HIV‐1,” Scandinavian Journal of Infectious Diseases 42, no. 5 (2010): 389–394. 20100115 10.3109/00365540903496536

[85] R. C. Su , A. Sivro , J. Kimani , W. Jaoko , F. A. Plummer , and T. B. Ball , “Epigenetic Control of IRF1 Responses in HIV‐Exposed Seronegative Versus HIV‐Susceptible Individuals,” Blood 117, no. 9 (2011): 2649–2657. 21200019 10.1182/blood-2010-10-312462 PMC3062355

[86] N. Nelson , Y. Kanno , and C. Hong , et al, “Expression of IFN Regulatory Factor Family Proteins in Lymphocytes. Induction of Stat‐1 and IFN Consensus Sequence Binding Protein Expression by T Cell Activation,” Journal of Immunology 156, no. 10 (1996): 3711–3720. 8621906

[87] R. C. Su , A. Plesniarski , Z. Ao , et al., “Reducing IRF‐1 to Levels Observed in HESN Subjects Limits HIV Replication, But not the Extent of Host Immune Activation,” Molecular Therapy. Nucleic Acids 4, no. 10 (2015): e259. 26506037 10.1038/mtna.2015.29 PMC4881757

[88] A. Sivro , R. C. Su , F. A. Plummer , and T. B. Ball , “HIV and Interferon Regulatory Factor 1: A Story of Manipulation and Control,” AIDS Research and Human Retroviruses 29, no. 11 (2013): 1428–1433. 23984938 10.1089/AID.2013.0098

[89] Y. Li , F. Lefebvre , E. Nakku‐Joloba , et al., “Upregulation of PTPRC and Interferon Response Pathways in HIV‐1 Seroconverters Prior to Infection,” Journal of Infectious Diseases 227, no. 5 (2023): 714–719. 36637125 10.1093/infdis/jiac498 PMC9978315

[90] A. C. Anderson , D. E. Anderson , L. Bregoli , et al., “Promotion of Tissue Inflammation by the Immune Receptor TIM‐3 Expressed on Innate Immune Cells,” Science 318, no. 5853 (2007): 1141–1143. 18006747 10.1126/science.1148536

[91] G. J. Freeman , J. M. Casasnovas , D. T. Umetsu , and R. H. DeKruyff , “TIM Genes: A Family of Cell Surface Phosphatidylserine Receptors That Regulate Innate and Adaptive Immunity,” Immunological Reviews 235, no. 1 (2010): 172–189. 20536563 10.1111/j.0105-2896.2010.00903.x PMC2914464

[92] L. Monney , C. A. Sabatos , J. L. Gaglia , et al., “Th1‐specific Cell Surface Protein Tim‐3 Regulates Macrophage Activation and Severity of an Autoimmune Disease,” Nature 415, no. 6871 (2002): 536–541. 11823861 10.1038/415536a

[93] R. B. Jones , L. C. Ndhlovu , J. D. Barbour , et al., “Tim‐3 Expression Defines a Novel Population of Dysfunctional T Cells With Highly Elevated Frequencies in Progressive HIV‐1 Infection,” The Journal of Experimental Medicine 205, no. 12 (2008): 2763–2779. 19001139 10.1084/jem.20081398 PMC2585847

[94] M. Sironi , M. Biasin , F. Gnudi , et al., “A Regulatory Polymorphism in HAVCR2 Modulates Susceptibility to HIV‐1 Infection,” PLoS One 9, no. 9 (2014): e106442. 25180498 10.1371/journal.pone.0106442 PMC4152274

[95] T. W. Chun , L. Carruth , D. Finzi , et al., “Quantification of Latent Tissue Reservoirs and Total Body Viral Load in HIV‐1 Infection,” Nature 387, no. 6629 (1997): 183–188. 9144289 10.1038/387183a0

[96] S. G. Deeks , “Shock and Kill,” Nature 487, no. 7408 (2012): 439–440. 22836995 10.1038/487439a

[97] A. J. Kleinman , R. Sivanandham , I. Pandrea , C. A. Chougnet , and C. Apetrei , “Regulatory T Cells As Potential Targets for HIV Cure Research,” Frontiers in Immunology 9 (2018): 734. 29706961 10.3389/fimmu.2018.00734 PMC5908895

[98] M. M. Painter , T. D. Zaikos , and K. L. Collins , “Quiescence Promotes Latent HIV Infection and Resistance to Reactivation From Latency With Histone Deacetylase Inhibitors,” Journal of Virology 91, no. 24 (2017): e01080‐17. 29021396 10.1128/JVI.01080-17 PMC5709582

[99] E. Bégaud , L. Chartier , V. Marechal , et al., “Reduced CD4 T Cell Activation and In Vitro Susceptibility to HIV‐1 Infection in Exposed Uninfected Central Africans,” Retrovirology 3, no. 1 (2006): 35. 16792805 10.1186/1742-4690-3-35 PMC1524799

[100] M. Biasin , S. L. Caputo , L. Speciale , et al., “Mucosal and Systemic Immune Activation is Present in Human Immunodeficiency Virus‐Exposed Seronegative Women,” Journal of Infectious Diseases 182, no. 5 (2000): 1365–1374. 11023460 10.1086/315873

[101] C. Tomescu , F. M. Duh , M. A. Lanier , et al., “Increased Plasmacytoid Dendritic Cell Maturation and Natural Killer Cell Activation in HIV‐1 Exposed, Uninfected Intravenous Drug Users,” AIDS 24, no. 14 (2010): 2151–2160. 20647906 10.1097/QAD.0b013e32833dfc20 PMC3253656

[102] C. Tomescu , K. E. Seaton , P. Smith , et al., “Innate Activation of MDC and NK Cells in High‐Risk HIV‐1–Exposed Seronegative IV‐Drug Users Who Share Needles When Compared With Low‐Risk Nonsharing IV‐Drug User Controls,” JAIDS Journal of Acquired Immune Deficiency Syndromes 68, no. 3 (2015): 264–273. 25514793 10.1097/QAI.0000000000000470 PMC4329050

[103] S. Akira , S. Uematsu , and O. Takeuchi , “Pathogen Recognition and Innate Immunity,” Cell 124, no. 4 (2006): 783–801. 16497588 10.1016/j.cell.2006.02.015

[104] K. Huik , R. Avi , M. Pauskar , et al., “Association between TLR3 rs3775291 and Resistance to HIV Among Highly Exposed Caucasian Intravenous Drug Users,” Infection, Genetics and Evolution: Journal of Molecular Epidemiology and Evolutionary Genetics in Infectious Diseases 20 (2013): 78–82. 23962581 10.1016/j.meegid.2013.08.008 PMC4001117

[105] M. Sironi , M. Biasin , R. Cagliani , et al., “A Common Polymorphism in TLR3 Confers Natural Resistance to HIV‐1 Infection,” Journal of Immunology 188, no. 2 (2012): 818–823. 10.4049/jimmunol.1102179 22174453

[106] C. Gorbea , K. A. Makar , M. Pauschinger , et al., “A Role for Toll‐Like Receptor 3 Variants in Host Susceptibility to Enteroviral Myocarditis and Dilated Cardiomyopathy,” Journal of Biological Chemistry 285, no. 30 (2010): 23208–23223. 20472559 10.1074/jbc.M109.047464 PMC2906314

[107] H. K. Tran , L. Chartier , L. X. Troung , et al., “Systemic Immune Activation in HIV‐1‐Exposed Uninfected Vietnamese Intravascular Drug Users,” AIDS Research and Human Retroviruses 22, no. 3 (2006): 255–261. 16545012 10.1089/aid.2006.22.255

[108] B. Walker and A. McMichael , “The T‐Cell Response to HIV,” Cold Spring Harbor Perspectives in Medicine 2, no. 11 (2012): a007054. 23002014 10.1101/cshperspect.a007054 PMC3543107

[109] S. M. Gonzalez , N. A. Taborda , and M. T. Rugeles , “Role of Different Subpopulations of CD8+ T Cells During HIV Exposure and Infection,” Frontiers in Immunology 8 (2017): 936. 28824656 10.3389/fimmu.2017.00936 PMC5545716

[110] V. Appay , D. F. Nixon , S. M. Donahoe , et al., “HIV‐Specific Cd8+ T Cells Produce Antiviral Cytokines But Are Impaired in Cytolytic Function,” The Journal of Experimental Medicine 192, no. 1 (2000): 63–76. 10880527 10.1084/jem.192.1.63 PMC1887711

[111] C. Restrepo , N. I. Rallón , J. del Romero , et al., “Low‐Level Exposure to HIV Induces Virus‐Specific T Cell Responses and Immune Activation in Exposed HIV‐Seronegative Individuals,” Journal of Immunology 185, no. 2 (2010): 982–989. 10.4049/jimmunol.1000221 20543099

[112] L. Flórez‐Álvarez , J. C. Hernandez , and W. Zapata , “NK Cells in HIV‐1 Infection: From Basic Science to Vaccine Strategies,” Frontiers in Immunology 9 (2018): 2290. 30386329 10.3389/fimmu.2018.02290 PMC6199347

[113] F. Borrego , M. J. Robertson , J. Ritz , J. Peña , and R. Solana , “CD69 Is a Stimulatory Receptor for Natural Killer Cell and Its Cytotoxic Effect is Blocked by CD94 Inhibitory Receptor,” Immunology 97, no. 1 (1999): 159–165. 10447727 10.1046/j.1365-2567.1999.00738.x PMC2326810

[114] S. R. Roff , E. N. Noon‐Song , and J. K. Yamamoto , “The Significance of Interferon‐γ in HIV‐1 Pathogenesis, Therapy, and Prophylaxis,” Frontiers in Immunology 4 (2014): 498. 24454311 10.3389/fimmu.2013.00498 PMC3888948

[115] N. Q. Zhao , R. Pi , D. N. Nguyen , et al., “NKp30 and NKG2D Contribute to Natural Killer Recognition of HIV‐Infected Cells,” bioRxiv 1 (2024): 600449. 10.1016/j.isci.2025.113548 PMC12514549 41079618

[116] M. Miyamoto , A. F. T. B. Gouvêa , E. Ono , R. C. M. Succi , S. Pahwa , and M. I. Moraes‐Pinto , “Immune Development in HIV‐Exposed Uninfected Children Born to HIV‐Infected Women,” Revista do Instituto de Medicina Tropical de São Paulo 59 (2017): e30. 28591258 10.1590/S1678-9946201759030 PMC5459537

[117] A. Mastrangelo , R. Banga , and M. Perreau , “Elite and Posttreatment Controllers, Two Facets of HIV Control,” Current Opinion in HIV and AIDS 17, no. 5 (2022): 325–332. 35938466 10.1097/COH.0000000000000751 PMC10004771

[118] R. A. Ahmed , K. O. Adekoya , C. K. Onwuamah , et al., “Mechanism of Viral Suppression Among HIV Elite Controllers and Long‐Term Nonprogressors in Nigeria and South Africa,” Viruses 14, no. 6 (2022): 1270. 35746741 10.3390/v14061270 PMC9228396

[119] M. López , V. Soriano , A. Peris‐Pertusa , N. Rallón , C. Restrepo , and J. M. Benito , “Elite Controllers Display Higher Activation on Central Memory CD8 T Cells Than HIV Patients Successfully on HAART,” AIDS Research and Human Retroviruses 27, no. 2 (2011): 157–165. 20964478 10.1089/aid.2010.0107

[120] D. G. Caetano , H. H. S. de Paula, , G. Bello , et al., “HIV‐1 Elite Controllers Present a High Frequency of Activated Regulatory T and Th17 Cells,” PLoS One 15, no. 2 (2020): e0228745. 32023301 10.1371/journal.pone.0228745 PMC7001932

[121] J. D. Barbour , L. C. Ndhlovu , Q. Xuan Tan , et al., “High CD8+ T Cell Activation Marks a Less Differentiated HIV‐1 Specific CD8+ T Cell Response That is Not Altered by Suppression of Viral Replication,” PLoS One 4, no. 2 (2009): e4408. 19198651 10.1371/journal.pone.0004408 PMC2634967

[122] J. Nilsson , A. Boasso , P. A. Velilla , et al., “HIV‐1–driven Regulatory T‐Cell Accumulation in Lymphoid Tissues Is Associated With Disease Progression in HIV/AIDS,” Blood 108, no. 12 (2006): 3808–3817. 16902147 10.1182/blood-2006-05-021576 PMC1895475

[123] P. W. Hunt , J. Brenchley , E. Sinclair , et al., “Relationship Between T Cell Activation and CD4+ T Cell Count in HIV‐Seropositive Individuals With Undetectable Plasma HIV RNA Levels in the Absence of Therapy,” Journal of Infectious Diseases 197, no. 1 (2008): 126–133. 18171295 10.1086/524143 PMC3466592

[124] V. D. Gonzalez , K. Falconer , K. G. Blom , et al., “High Levels of Chronic Immune Activation in the T‐Cell Compartments of Patients Coinfected With Hepatitis C Virus and Human Immunodeficiency Virus Type 1 and on Highly Active Antiretroviral Therapy are Reverted by Alpha Interferon and Ribavirin Treatment,” Journal of Virology 83, no. 21 (2009): 11407–11411. 19710147 10.1128/JVI.01211-09 PMC2772767

[125] E. S. Jacobs , S. M. Keating , M. Abdel‐Mohsen , et al., “Cytokines Elevated in HIV Elite Controllers Reduce HIV Replication In Vitro and Modulate HIV Restriction Factor Expression,” Journal of Virology 91, no. 6 (2017): e02051–16. 28053103 10.1128/JVI.02051-16 PMC5331794

[126] F. Z. Chowdhury , Z. Ouyang , M. Buzon , B. D. Walker , M. Lichterfeld , and X. G. Yu , “Metabolic Pathway Activation Distinguishes Transcriptional Signatures of CD8+ T Cells From HIV‐1 Elite Controllers,” AIDS 32, no. 18 (2018): 2669–2677. 30289807 10.1097/QAD.0000000000002007 PMC6317861

[127] C. M. Card , Y. Keynan , J. Lajoie , et al., “HIV Controllers Are Distinguished by Chemokine Expression Profile and HIV‐Specific T‐Cell Proliferative Potential,” JAIDS Journal of Acquired Immune Deficiency Syndromes 59, no. 5 (2012): 427–437. 22240463 10.1097/QAI.0b013e3182454fcd

[128] H. Hocini , H. Bonnabau , C. Lacabaratz , et al., “HIV Controllers Have Low Inflammation Associated With a Strong HIV‐Specific Immune Response in Blood,” Journal of Virology 93, no. 10 (2019): e01690–18. 30814287 10.1128/JVI.01690-18 PMC6498051

[129] B. Emu , E. Sinclair , D. Favre , et al., “Phenotypic, Functional, and Kinetic Parameters Associated With Apparent T‐Cell Control of Human Immunodeficiency Virus Replication in Individuals With and Without Antiretroviral Treatment,” Journal of Virology 79, no. 22 (2005): 14169–14178. 16254352 10.1128/JVI.79.22.14169-14178.2005 PMC1280210

[130] R. Sachdeva , R. Y. Shilpi , and M. Simm , “The Interplay Between the X‐DING‐CD4, IFN‐α and IL‐8 Gene Activity in Quiescent and Mitogen or HIV‐1 Exposed Pbmcs From HIV‐1 Elite Controllers, AIDS Progressors and HIV Negative Controls,” Innate Immunity 20, no. 2 (2014): 173–183. 23751822 10.1177/1753425913486162 PMC3883920

[131] R. Sachdeva , Y. Li , R. Y. Shilpi , and M. Simm , “Human X‐DING‐CD4 Mediates Resistance to HIV‐1 Infection Through Novel Paracrine‐Like Signaling,” FEBS journal marzo de 282, no. 5 (2015): 937–950. 25581464 10.1111/febs.13192 PMC4351123

[132] M. Singh , S. M. Leddy , L. P. Iñiguez , M. L. Bendall , D. F. Nixon , and C. Feschotte , “Transposable Elements May Enhance Antiviral Resistance in HIV‐1 Elite Controllers,” bioRxiv 12 (2023): 571123. 10.1186/s13059-025-03484-y PMC11849351 39988678

