# Correction to “Natural Resistance to HIV Infection: Role of Immune Activation”

**DOI:** 10.1002/iid3.70526

**Published:** 2026-09-10

Naranjo‐Covo, M.M., Rincón‐Tabares, D.S., Flórez‐Álvarez, L., Hernandez, J.C. and Zapata‐Builes, W. (2025), Natural Resistance to HIV Infection: Role of Immune Activation. Immunity, Inflammation and Disease, 13: e70138. https://doi.org/10.1002/iid3.70138

The affiliations of the authors “Wildeman Zapata‐Builes” and “Juan C. Hernandez” were incorrectly linked to affiliations 1 and 2. Their correct affiliation is only number 2 as follows:

“Grupo Infettare, Facultad de Medicina, Universidad Cooperativa de Colombia, Medellín, Colombia.”

The online version of this article has been corrected accordingly

We apologize for this error.

